# Genome-wide association study of preserved ratio impaired spirometry (PRISm)

**DOI:** 10.1183/13993003.00337-2023

**Published:** 2024-01-04

**Authors:** Daniel H. Higbee, Alvin Lirio, Fergus Hamilton, Raquel Granell, Annah B. Wyss, Stephanie J. London, Traci M. Bartz, Sina A. Gharib, Michael H. Cho, Emily Wan, Edwin Silverman, James D. Crapo, Jesus V.T. Lominchar, Torben Hansen, Niels Grarup, Thomas Dantoft, Line Kårhus, Allan Linneberg, George T. O'Connor, Josée Dupuis, Hanfie Xu, Maaike M. De Vries, Xiaowei Hu, Stephen S. Rich, R. Graham Barr, Ani Manichaikul, Sara R.A. Wijnant, Guy G. Brusselle, Lies Lahousse, Xuan Li, Ana I. Hernández Cordero, Ma'en Obeidat, Don D. Sin, Sarah E. Harris, Paul Redmond, Adele M. Taylor, Simon R. Cox, Alexander T. Williams, Nick Shrine, Catherine John, Anna L. Guyatt, Ian P. Hall, George Davey Smith, Martin D. Tobin, James W. Dodd

**Affiliations:** 1 MRC Integrative Epidemiology Unit (IEU), University of Bristol, Bristol, UK; 2 Academic Respiratory Unit, University of Bristol, Southmead Hospital, Bristol, UK; 3 Department of Population Health Sciences, University of Leicester, Leicester, UK; 4 Epidemiology Branch, National Institute of Environmental Health Sciences, National Institutes of Health, Research Triangle Park, NC, USA; 5 Cardiovascular Health Research Unit, Departments of Biostatistics and Medicine, University of Washington, Seattle, WA, USA; 6 Computational Medicine Core, Center for Lung Biology, UW Medicine Sleep Center, Department of Medicine, University of Washington, Seattle, WA, USA; 7 Channing Division of Network Medicine, Brigham and Women's Hospital, Boston, MA, USA; 8 Harvard Medical School, Boston, MA, USA; 9 Pulmonary and Critical Care Section, Department of Medicine, VA Boston Healthcare System, Boston, MA, USA; 10 Division of Pulmonary, Critical Care and Sleep Medicine, National Jewish Health, Denver, CO, USA; 11 Novo Nordisk Foundation Center for Basic Metabolic Research, Faculty of Health and Medical Sciences, University of Copenhagen, Copenhagen, Denmark; 12 Center for Clinical Research and Prevention, Bispebjerg and Frederiksberg Hospital, Frederiksberg, Denmark; 13 Pulmonary Center, School of Medicine, Boston University, Boston, MA, USA; 14 Division of Pulmonary, Allergy, Sleep, and Critical Care Medicine, Boston Medical Center, Boston, MA, USA; 15 Department of Epidemiology, Biostatistics and Occupational Health, McGill University, Montreal, QC, Canada; 16 Department of Biostatistics, Boston University School of Public Health, Boston, MA, USA; 17 Department of Epidemiology, University of Groningen, University Medical Center Groningen, Groningen, The Netherlands; 18 Groningen Research Institute for Asthma and COPD, University of Groningen, University Medical Center Groningen, Groningen, The Netherlands; 19 Center for Public Health Genomics, University of Virginia, Charlottesville, VA, USA; 20 Department of Medicine, College of Physicians and Surgeons, Columbia University, New York, NY, USA; 21 Department of Bioanalysis, Ghent University, Ghent, Belgium; 22 Department of Epidemiology, Erasmus MC, Rotterdam, The Netherlands; 23 Department of Respiratory Medicine, Ghent University Hospital, Ghent, Belgium; 24 Department of Epidemiology, Department of Respiratory Medicine, Erasmus MC, Rotterdam, The Netherlands; 25 Centre for Heart Lung Innovation, University of British Columbia, St. Paul's Hospital, Vancouver, BC, Canada; 26 Division of Respiratory Medicine, Department of Medicine, University of British Columbia, Vancouver, BC, Canada; 27 Lothian Birth Cohorts, Department of Psychology, University of Edinburgh, Edinburgh, UK; 28 University of Nottingham and NIHR Nottingham Biomedical Research Centre, Nottingham, UK; 29 Leicester NIHR Biomedical Research Centre, Leicester, UK; 30 Joint senior authors

## Abstract

**Background:**

Preserved ratio impaired spirometry (PRISm) is defined as a forced expiratory volume in 1 s (FEV_1_) <80% predicted and FEV_1_/forced vital capacity ≥0.70. PRISm is associated with respiratory symptoms and comorbidities. Our objective was to discover novel genetic signals for PRISm and see if they provide insight into the pathogenesis of PRISm and associated comorbidities.

**Methods:**

We undertook a genome-wide association study (GWAS) of PRISm in UK Biobank participants (Stage 1), and selected single nucleotide polymorphisms (SNPs) reaching genome-wide significance for replication in 13 cohorts (Stage 2). A combined meta-analysis of Stage 1 and Stage 2 was done to determine top SNPs. We used cross-trait linkage disequilibrium score regression to estimate genome-wide genetic correlation between PRISm and pulmonary and extrapulmonary traits. Phenome-wide association studies of top SNPs were performed.

**Results:**

22 signals reached significance in the joint meta-analysis, including four signals novel for lung function. A strong genome-wide genetic correlation (r_g_) between PRISm and spirometric COPD (r_g_=0.62, p<0.001) was observed, and genetic correlation with type 2 diabetes (r_g_=0.12, p=0.007). Phenome-wide association studies showed that 18 of 22 signals were associated with diabetic traits and seven with blood pressure traits.

**Conclusion:**

This is the first GWAS to successfully identify SNPs associated with PRISm. Four of the signals, rs7652391 (nearest gene *MECOM*), rs9431040 (*HLX*), rs62018863 (*TMEM114*) and rs185937162 (*HLA-B*), have not been described in association with lung function before, demonstrating the utility of using different lung function phenotypes in GWAS. Genetic factors associated with PRISm are strongly correlated with risk of both other lung diseases and extrapulmonary comorbidity.

## Tweetable abstract

**This is the first GWAS to report genome-wide significant SNPs for PRISm, four of which are novel for lung function. Genetic factors associated with PRISm are strongly correlated with risk of both other lung diseases and extrapulmonary comorbidity.** https://bit.ly/3Qo0jUn

## Introduction

Preserved ratio impaired spirometry (PRISm), also referred to as “restrictive pattern” or “unclassified” spirometry, is defined as forced expiratory volume in 1 s (FEV_1_) <80% predicted and FEV_1_/forced vital capacity (FVC) ratio ≥0.70 [1]. It has been suggested that for a subgroup of subjects, PRISm may be a precursor of COPD, with up to 50% progressing to COPD while 15% return to “normal” spirometry over 5 years [2, 3]. A larger and younger cohort has shown that PRISm may be transient with only 12% going on to develop airflow obstruction over 8 years [4]. Clinical interest in PRISm relates to its consistent association with respiratory symptoms, comorbidities (*e.g.* obesity, diabetes and cardiovascular disease) and all-cause mortality [2–4].

Previous studies have shown that lung function measures or traits are, in part, heritable and associated with genetic variants, implicating a wide range of mechanisms including cilia development and elastic fibres in obstructive lung disease [5, 6], but the individual genetic associations and pathways which underlie PRISm are less well understood. A previous genome-wide association study (GWAS) of PRISm failed to find associations of genome-wide significance (p<5×10^−8^), was modest, and was restricted to ever-smokers [7].

Genetic variants associated with PRISm could provide invaluable insight into its pathogenesis and associated comorbidities, as well as potentially identify therapeutic targets.

Our objective was to perform a case–control GWAS of PRISm and report novel associated single nucleotide polymorphisms (SNPs) in a two-stage study design see if they provide insight into the pathogenesis of PRISm and associated comorbidities.

## Methods

### Study design

We performed a two-stage GWAS with meta-analysis. For the discovery cohort (Stage 1), we used the UK Biobank (UKBB) (www.ukbiobank.ac.uk). For Stage 2 we used cohorts within the SpiroMETA and Cohort for Heart and Aging Research in Genomic Epidemiology (CHARGE) consortia as well as COPDGene. We estimated genetic correlations with potentially related phenotypic traits. We performed a phenome-wide association study (PheWAS) of SNPs not found to be associated with lung function in the largest lung function GWAS to date [8].

### Stage 1

**FIGURE 1:**
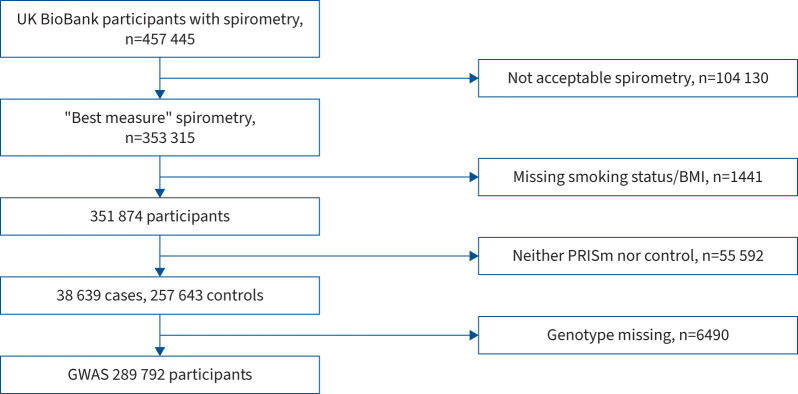
Participant selection flow chart. BMI: body mass index; PRISm: preserved ratio impaired spirometry; GWAS: genome-wide association study.

The UKBB, which was used for the Stage 1 GWAS, is a large UK population-based health research resource of ∼500 000 people aged 38–73 years old recruited between 2006 and 2010. Questionnaires, interviews, anthropometric measures and biological samples were collected. UKBB received ethical approval from the Research Ethics Committee (REC reference for UK Biobank: 11/NW/0382). We used previously derived variables of quality-controlled prebronchodilator FEV_1_ and FVC. Only participants with spirometry classified as acceptable were included (supplementary appendix 1). Only those of self-identified European ancestry with very similar genetic ancestry based on principal component analysis of genotypes were included. Patients with unknown smoking status or weight were excluded. FEV_1_ % predicted was calculated as per Global Lung Function Initiative (GLI) 2012 values using RSpiro R package in R v3.6.1 (www.r-project.org). PRISm was defined as FEV_1_ <80% predicted and FEV_1_/FVC ratio ≥0.70 and controls as FEV_1_ ≥80% predicted and FEV_1_/FVC ratio ≥0.70. Participants with spirometry not meeting the criteria for PRISm or control were excluded. Figure 1 shows the participant selection flow chart and table 1 contains demographics of the sample used.

**TABLE 1:** Demographics of Stage 1 participants in UK Biobank

**Demographic at baseline**	**PRISm**	**Controls**
**Participants (n)**	38 639	257 643
**Age (years)**	56.4±7	56.0±7
**BMI (kg·m^−2^)**	29.1±5	27.2±4
**Female (%)**	55.4	55.6
**FEV_1_ (% predicted)**	74 (68–77)	98 (90–106)
**FVC (% predicted)**	76 (71–81)	99 (91–108)
**FEV_1_/FVC**	0.75 (0.72–0.78)	0.77 (0.74–0.80)
**Never-smoker (%)**	51.2	56.8
**Ex-smoker (%)**	36.4	35.3
**Current smoker (%)**	12.4	7.9
**Pack-years** ^#^	23 (13–36)	16 (8–27)
**Doctor-diagnosed asthma (%)**	16.8	9.9
**Doctor-diagnosed COPD (%)**	1.7	0.3

Data are presented as mean±sd or median (interquartile range), unless otherwise stated. PRISm: preserved ratio impaired spirometry; BMI: body mass index; FEV_1_: forced expiratory volume in 1 s; FVC: forced vital capacity. ^#^: for current and ex-smokers only.

The GWAS was performed using the Integrative Epidemiology Unit (IEU) GWAS pipeline, described in detail elsewhere [9]. The pipeline contains previously derived genetic files of participants after pre-imputation quality control, phasing and imputation, allowing fast standardised GWAS of the UKBB population. A description of the process to create the derived genetic files can be found in detail elsewhere [10, 11] and can be summarised as follows. Genotyping was performed using the Axiom UK BiLEVE array and the Axiom Biobank array (Affymetrix) [12]. Before phasing, multiallelic SNPs or those with minor allele frequency (MAF) ≤1% were removed. Phasing of genotype data was performed using a modified version of the SHAPEIT2 algorithm [13]. Genotype imputation to a reference set combining the UK10K haplotype and Haplotype Reference Consortium reference panels was performed using IMPUTE2 algorithms [14]. The analyses were restricted to autosomal variants using graded filtering with varying imputation quality for different allele frequency ranges. An in-house algorithm was then applied to preferentially remove the individuals related to the greatest number of other individuals until no related pairs remain. To model population structure in the sample, 143 006 directly genotyped SNPs were used, obtained after omitting variants with MAF <0.01, genotyping missing rate >0.015 or Hardy–Weinberg equilibrium p<0.0001; and linkage disequilibrium (LD) pruning to an r^2^ threshold of 0.1 using PLINK v2.00 (www.cog-genomics.org/plink/2.0).

Using the pipeline-derived genetic files, a GWAS of PRISm *versus* controls was performed using BOLT-LMM (https://alkesgroup.broadinstitute.org/BOLT-LMM/BOLT-LMM_manual.html) [15]. The association between PRISm and each SNP was calculated using logistic regression, with SNP coded additively, and adjusting for sex, body mass index (BMI), age and smoking status (smoking status as a dummy variable; 0=never-smoker, 1=ex-smoker, 2=current smoker).

**FIGURE 2:**
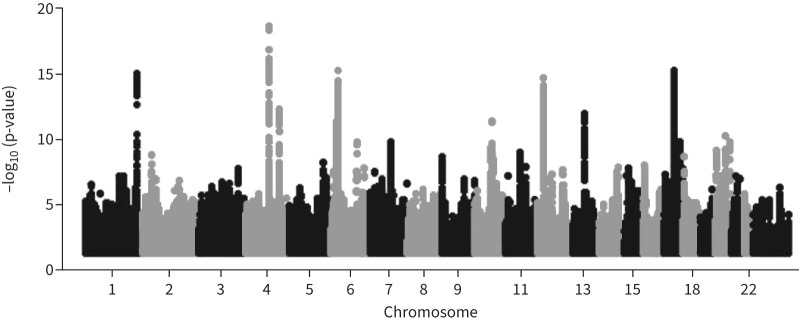
Manhattan plot of discovery genome-wide association study after linkeage disequilibrium score regression filtering and adjustment.

SNPs were filtered to remove those with a MAF ≤0.01 or that were strand-ambiguous. LD score regression was used to estimate heritability and to assess genomic inflation by calculating λ and LD intercept [16]. To correct for genomic inflation the p-values were corrected for the LD intercept. Stringent LD clumping (r^2^=0.001, kb 10 000) was applied to SNPs reaching a significance threshold of p=5×10^−8^ to define distinct sentinel SNPs. Only SNPs considered novel, based on their reference SNP cluster IDs (rsIDs) not being reported as top signals in the Shrine *et al*. [6] 2019 GWAS of lung function, were investigated in the replicating cohorts. Figure 2 shows a flow chart of the analysis.

### Stage 2 and joint analysis

Novel SNPs identified in Stage 1 were tested for association in 13 European ancestry independent cohorts from the SpiroMeta and CHARGE consortia. The supplementary materials summarise full cohort descriptions, spirometry methods, genotyping methods and imputation platforms. Replicating cohorts performed a logistic regression with the lead SNPs from Stage 1 in those with PRISm and control spirometry. Adjustment was made for age, BMI, sex, smoking history (either pack-years or status, as described above) and population substructure by either principal components or using linear mixed models [17]. Results were combined across the Stage 2 studies using a fixed-effect inverse variance model in Stata 17 (StatCorp., College Station, TX, USA). The nearest gene for each SNP was determined using PhenoScanner (www.phenoscanner.medschl.cam.ac.uk).

### Definition of top SNPs

We performed a joint analysis of Stage 1 and Stage 2 in a fixed-effect inverse variance model using Stata 17. Heterogeneity was tested for using Cochrane Q. Top SNPs had to meet the following criteria: p<5×10^−8^ in the joint analysis of Stage 1+2; same direction of effect in Stage 1 and Stage 2; and either p<0.05 in Stage 2 or a lower p-value in the joint Stage 1+2 than in Stage 1. Because no genome-wide significant SNPs have ever been reported in association with PRISm, all top SNPs identified are assumed novel for PRISm.

### Genetic correlation

To investigate the shared genetic architecture between PRISm and other traits, we performed a bivariate LD score regression analysis to assess the genome-wide genetic correlation (r_g_) between Stage 1 PRISm results and continuous lung function traits, moderate to severe asthma, asthma–COPD overlap, spirometrically diagnosed COPD, respiratory tract infections and eosinophil count [18]. We also examined the genetic correlation between PRISm and related conditions including type 2 diabetes, BMI, hypertension and myocardial infarction.

### PheWAS

We conducted a PheWAS of each top SNP individually using https://gwas.mrcieu.ac.uk/phewas/ to determine if SNP pleiotropy could account for the described associations with PRISm. We highlight lung function measures, lung diseases and comorbidities previously associated with PRISm (*e.g.* overweight or BMI, cardiovascular disease and diabetes) [4].

### SNPs novel for lung function: deep PheWAS and gene ontology

Top SNPs that were not reported in the largest GWAS of lung function to date (r^2^≥0.5) were reported as novel for both PRISm and lung function [8]. The SNPs were analysed using deep PheWAS enriched for lung function traits to examine for associations with lung function and other traits [19]. The nearest genes to these novel SNPs were then investigated for gene ontology information using the Functional Mapping and Annotation of Genome-wide association studies (FUMA) tool (https://fuma.ctglab.nl). Hypergeometric tests were implemented using FUMA to test whether the nearest genes to novel SNPs were overrepresented in gene sets from MSigDB, WikiPathways and reported genes from the GWAS catalogue (gene-set enrichment analysis; see https://fuma.ctglab.nl/tutorial#gene2func for full list of gene sets) [20].

## Results

### Discovery GWAS

FEV_1_ and FVC “best measures” were available for 353 315 UKBB participants. Supplementary appendix 1 contains details of spirometric quality control to derive best measure variables. After excluding individuals missing smoking status and/or BMI (n=1441), 38 639 PRISm cases and 257 643 controls were identified.

After further excluding 6490 individuals without derived genetic files in the GWAS pipeline described above, a GWAS of 289 792 individuals was performed (figure 1). A total of 7 339 387 SNPs were tested after exclusions. A Manhattan plot of the results is shown in figure 2. The chip heritability estimate (h2) with se was 0.0493±0.0024. The λ was 1.25 and the LD intercept 1.02. The 7 339 378 SNP p-values were corrected for the LD intercept, leaving 6037 that met p<5×10^−8^.

After LD clumping (r^2^=0.001, kb 10 000), 33 SNPs from 18 chromosomes remained. We removed SNPs already described in the Shrine *et al.* [6] GWAS, leaving 27 SNPs from 16 chromosomes to investigate in Stage 2.

### Stage 2 analysis

**FIGURE 3:**
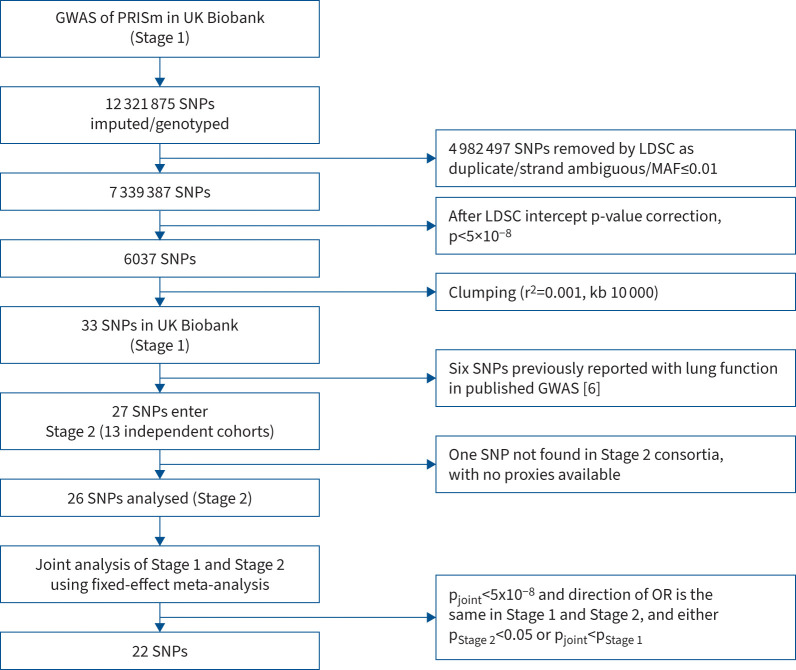
Flow chart of single nucleotide polymorphisms (SNPs) analysed. GWAS: genome-wide association study; PRISm: preserved ratio impaired spirometry; LDSC: linkeage disequilibrium score regression; MAF: minor allele frequency; OR: odds ratio.

The Stage 2 analysis to replicate SNPs discovered in Stage 1 was conducted in 13 cohorts (5165 PRISm cases and 47 729 controls). Stage 2 cohorts used proxies if SNPs were not found in their panel (r^2^≥0.8). SNP rs142330941 was only found in the Framingham cohort and no proxies for it were available in other cohorts, so it was excluded from further analysis, leaving 26 SNPs tested in Stage 2 (figure 3).

In total, 22 SNPs met the criteria for top SNPs. Five of these showed strong evidence of heterogeneity contributing to effect with Cochrane Q p<0.05 and I^2^≥40%. The large difference of sample sizes of Stage 1 and 2 likely contributed to this heterogeneity. Full results at each stage are in table 2.

**TABLE 2:** SNP results Stage 1, Stage 2 and joint Stage 1+Stage 2

**rsID**	**CHR:BP**	**Nearest gene**	**Function**	**EA/NEA**	**EAF**	**Stage 1**		**Stage 2**		**Stage 1+Stage 2 joint meta-analysis**			
						**OR (95% CI)**	**p-value**	**OR (95% CI)**	**p-value**	**OR (95% CI)**	**p-value**	**I^2^ (%)**	**Cochrane Q p-value**
**rs9431040**	**1:221152299**	***HLX***	**Intergenic**	**T/C**	**0.28**	**0.99 (0.990–0.994)**	**1.88×10^−15^**	**0.94 (0.901–0.990)**	**0.020**	**0.99 (0.990–0.994)**	**4.56×10^−16^**	**0.0**	**0.74**
rs9295	2:36777825	*CRIM1*	UTR3	G/A	0.71	1.01 (1.004–0.008)	2.72×10^−9^	0.97 (0.923–1.014)	0.175	1.01 (1.004–1.008)	2.61×10^−9^	3.6	0.41
**rs7652391**	**3:168913273**	***MECOM***	**Intron**	**G/T**	**0.78**	**0.99 (0.992–0.996)**	**2.29×10^−8^**	**0.95 (0.902–1.007)**	**0.076**	**0.99 (0.992–0.996)**	**1.10×10^−8^**	**4.8**	**0.40**
**rs6537297**	**4:145502029**	***FHDC1***	**Intergenic**	**C/T**	**0.45**	**1.01 (1.005–1.008)**	**7.73×10^−13^**	**1.05 (1.003–1.093)**	**0.040**	**1.01 (0.996–1.016)**	**2.49×10^−13^**	**43.7**	**0.03**
**rs6923462**	**6:7801112**	***BMP6***	**Intron**	**T/C**	**0.84**	**0.99 (0.991–0.996)**	**4.06×10^−8^**	**0.91 (0.860–0.967)**	**0.002**	**0.99 (0.991–0.995)**	**1.45×10^−8^**	**40.7**	**0.04**
**rs1233604**	**6:28734676**	***ZFP57***	**Intergenic**	**G/A**	**0.88**	**0.99 (0.986–0.992)**	**1.18×10^−15^**	**1.00 (0.929–1.074)**	**0.978**	**0.99 (0.985–0.993)**	**5.96×10^−16^**	**0.0**	**0.61**
**rs185937162**	**6:31325268**	***HLA-B***	**Upstream**	**T/G**	**0.96**	**0.99 (0.984–0.992)**	**3.69×10^−8^**	**0.95 (0.326–2.760)**	**0.339**	**0.99 (0.984–0.992)**	**2.16×10^−8^**	**13.7**	**0.29**
**rs6928024**	**6:142551082**	***AL356739.1***	**Intergenic**	**G/A**	**0.75**	**1.01 (1.004–1.008)**	**2.44×10^−8^**	**1.03 (0.626–1.700)**	**0.226**	**1.01 (1.004–1.008)**	**1.34×10^−8^**	**39.9**	**0.05**
**rs10278266**	**7:14943333**	***DGKB***	**Intron**	**A/G**	**0.79**	**0.99 (0.992–0.996)**	**3.49×10^−8^**	**0.92 (0.875–0.976)**	**0.005**	**0.99 (0.992–0.996)**	**1.36×10^−8^**	**23.0**	**0.19**
**rs9649071**	**7:84524701**	***HMGN2P11***	**Intergenic**	**A/G**	**0.75**	**1.01 (1.004–1.008)**	**2.36×10^−10^**	**1.00 (0.949–1.047)**	**0.915**	**1.01 (1.004–1.008)**	**1.59×10^−10^**	**23.9**	**0.18**
rs7853305	9:4132402	*GLIS3*	Intron	G/C	0.61	0.99 (0.993–0.996)	1.00×10^−9^	0.96 (0.924–1.007)	0.106	1.00 (0.993–0.997)	1.46×10^−9^	6.3	0.38
**rs780151**	**10:80931481**	***ZMIZ1***	**Intron**	**G/A**	**0.58**	**1.01 (1.003–1.007)**	**5.11×10^−9^**	**1.07 (1.021–1.113)**	**0.004**	**1.01 (1.003–1.007)**	**1.79×10^−9^**	**42.5**	**0.03**
**rs12808829**	**11:62378660**	***EML3***	**Synonymous**	**G/A**	**0.63**	**1.01 (1.004–1.007)**	**1.35×10^−9^**	**1.05 (1.006–1.101)**	**0.025**	**1.01 (1.004–1.008)**	**5.25×10^−10^**	**42.5**	**0.03**
rs79487293	12:65905126	*RP11-230G5.2*	Intron	C/T	0.68	0.99 (0.993–0.099)	4.09×10^−8^	0.98 (0.604–1.611)	0.595	0.99 (0.993–0.997)	4.45×10^−8^	0.0	0.54
**rs11113217**	**12:107597518**	***SETP7***	**Intergenic**	**T/C**	**0.38**	**0.99 (0.993–0.997)**	**2.40×10^−8^**	**0.94 (0.902–0.988)**	**0.012**	**0.99 (0.993–0.997)**	**1.39×10^−8^**	**24.6**	**0.17**
rs7326916	13:71700945	*LINC00348*	Intron	A/T	0.39	1.01 (1.005–1.008)	9.20×10^−13^	1.01 (0.648–1.559)	0.817	1.01 (1.004–1.008)	1.01×10^−12^	0.0	0.52
**rs11623779**	**14:93096391**	***RIN3***	**Intron**	**T/C**	**0.82**	**1.01 (1.004–1.008)**	**3.58×10^−8^**	**1.04 (0.587–1.836)**	**0.199**	**1.01 (1.004–1.008)**	**1.95×10^−8^**	**1.2**	**0.44**
**rs11621083**	**14:102559538**	***HSP90AA1***	**Intron**	**T/A**	**0.15**	**0.99 (0.991–0.995)**	**1.91×10^−8^**	**0.97 (0.537–1.740)**	**0.252**	**0.99 (0.991–0.995)**	**1.04×10^−8^**	**0.0**	**0.56**
**rs1717198**	**15:41465862**	***EXD1***	**Intergenic**	**T/C**	**0.56**	**1.00 (1.003–1.007)**	**2.72×10^−8^**	**1.02 (0.665–1.575)**	**0.291**	**1.01 (1.003–1.007)**	**1.54×10^−8^**	**0.0**	**0.62**
**rs2240885**	**16:3647098**	***SLX4***	**Intron**	**G/A**	**0.78**	**0.99 (0.992–0.996)**	**1.35×10^−8^**	**0.94 (0.546–1.611)**	**0.018**	**0.99 (0.992–0.996)**	**5.57×10^−9^**	**3.1**	**0.42**
**rs62018863**	**16:8624118**	***TMEM114***	**Upstream**	**G/A**	**0.88**	**0.99 (0.990–0.995)**	**3.10×10^−8^**	**0.96 (0.479–1.904)**	**0.183**	**0.99 (0.990–0.994)**	**1.65×10^−8^**	**2.5**	**0.42**
**rs139077859**	**17:44335579**	***RP11-259G18.3***	**Downstream**	**G/A**	**0.79**	**0.99 (0.989–0.993)**	**1.01×10^−15^**	**0.93 (0.459–1.896)**	**0.056**	**0.99 (0.989–0.993)**	**3.14×10^−16^**	**0.0**	**0.50**
**rs11651469**	**17:69148519**	***CASC17***	**Intron**	**T/G**	**0.44**	**0.99 (0.993–0.996)**	**2.42×10^−10^**	**0.89 (0.698–0.908)**	**2.6×10^−23^**	**0.99 (0.992–0.996)**	**1.22×10^−12^**	**87.1**	**0.00**
**rs1000972**	**20:6621717**	***RP5-971N18.3***	**Intergenic**	**G/A**	**0.36**	**1.01 (1.004–1.007)**	**1.08×10^−9^**	**1.04 (0.660–1.625)**	**0.127**	**1.01 (1.004–1.008)**	**4.98×10^−10^**	**0.0**	**0.56**
**rs3091552**	**20:45440006**	***AL031055.1***	**Upstream**	**C/G**	**0.27**	**0.99 (0.991–0.995)**	**4.60×10^−12^**	**0.96 (0.913–1.01)**	**0.121**	**0.99 (0.991–0.995)**	**1.73×10^−12^**	**20.1**	**0.22**
**rs55791529**	**20:62363858**	***ZGPAT***	**Intron**	**C/T**	**0.33**	**0.99 (0.992–0.996)**	**2.67×10^−10^**	**0.95 (0.594–1.521)**	**0.032**	**0.99 (0.992–0.996)**	**1.01×10^−10^**	**27.0**	**0.15**

Gene was determined using PhenoScanner (https://www.phenoscanner.medschl.cam.ac.uk/). SNPs reaching criteria for top signals (p_joint_<5×10^−8^ *and* direction of odds ratio is the same in Stage 1 and Stage 2, *and* either p_stage 2_<0.05 *or* p_joint_<p_stage 1_) are highlighted in bold. SNP: single nucleotide polymorphism; CHR:BP: chromosome:base pair position; EA: effect allele; NEA: non-effect allele; EAF: allele 1 frequency.

### Genetic correlation

Details of GWAS used for correlation studies with the Stage 1 PRISm can be found in supplementary appendix 2. As expected, we found very strong genetic correlation between PRISm and FEV_1_ and FVC, and a gradation of increasing genetic correlation between PRISm and asthma, asthma–COPD overlap and COPD (table 3). Type 2 diabetes showed a moderate genetic correlation with PRISm. Waist-to-hip ratio (after adjustment for BMI) was positively genetically correlated with PRISm, whereas BMI was negatively genetically correlated with PRISm. Cardiac diseases, systolic hypertension and myocardial infarction showed positive genetic correlations with PRISm.

**TABLE 3:** Genetic correlation between PRISm and pulmonary and extrapulmonary traits

**Trait**	**r_g_±se**	**p-value**
**FEV_1_**	−0.96±0.01	<0.001
**FVC**	−0.93±0.01	<0.001
**PEFR**	−0.65±0.02	<0.001
**FEV_1_/FVC**	−0.23±0.02	<0.001
**COPD**	0.62±0.03	<0.001
**Asthma–COPD overlap**	0.52±0.04	<0.001
**Moderate to severe asthma**	0.31±0.05	<0.001
**Respiratory infection**	0.18±0.6	0.003
**Blood eosinophils**	0.06±0.02	0.012
**Type 2 diabetes**	0.12±0.03	0.007
**BMI**	−0.04±0.02	0.031
**Waist-to-hip-ratio^#^**	0.12±0.02	<0.001
**Systolic hypertension**	0.08±0.02	<0.001
**Diastolic hypertension**	0.05±0.02	0.035
**Myocardial infarction**	0.07±0.03	0.007

PRISm: preserved ratio impaired spirometry; r_g_: genome-wide genetic correlation; FEV_1_: forced expiratory volume in 1 s; FVC: forced vital capacity; PEFR: peak expiratory flow rate; BMI: body mass index. ^#^: adjusted for BMI.

### PheWAS

Almost all SNPs had associations with lung function traits (21 of 22 top SNPs). Consistent with the genetic correlation estimates, many SNPs were associated with anthropomorphic traits such as height (12 of 22) and weight or BMI (16 of 22). Associations with diabetes (diagnosis or medication use) or HbA1c (18 of 22) and systolic or diastolic pressure (7 of 22) were common. Full results are in the supplementary tables.

### SNPs novel for lung function: deep PheWAS and gene ontology

During the development of this paper, the largest GWAS of lung function was released that describes 1020 SNPs associated with lung function [8]. Four of the PRISm top SNPs were distinct from lung function signals found in this GWAS (r^2^>0.5) and are therefore novel for lung function: rs7652391 (nearest gene *MECOM*), rs9431040 (*HLX*), rs62018863 (*TMEM114*) and rs185937162 (*HLA-B*). We selected these four SNPs for a deep PheWAS enriched for lung function traits. All four SNPs were associated with lung function traits. This is expected because the deep PheWAS used UK Biobank as did our Stage 1 analysis. Rs9431040 was positively associated with systolic blood pressure (supplementary figure E4). As shown in supplementary figure E5, rs185937162 had a strong negative association with inflammatory conditions such as ankylosing spondylitis, uveitis and other spondyloarthropathies. For full results see supplementary tables and figures. Using FUMA, *HLA-B*, *HLX* and, to a lesser extent, *MECOM* were all shown to be expressed in the lung (HLA-B is expressed in nearly all cell types) (supplementary figure E6). *MECOM* encodes a protein that is a transcriptional regulator and oncoprotein and may be involved in haematopoiesis, apoptosis, development and cell differentiation and proliferation. As per the GeneCards database (www.genecards.org), SNPs in the gene have been associated with changes in body height and diastolic blood pressure. *HLX* is predicted to be involved in organ development and is associated with diseases affecting the diaphragm. SNPs in the gene are associated with changes in body height and cholesterol levels. *TMEM114* encodes a protein that has a role in lens and eye development. SNPs in the gene are associated with changes in vital capacity, body height and smoking initiation. *HLA-B* plays a role in the immune system and may influence the susceptibility to infection or the effect of autoimmune processes on the lung. Gene-set enrichment analyses showed that, in addition to respiratory traits, the 26 novel genes were enriched among gene sets for multiple other phenotypes, including white blood cell traits, anthropometric traits and traits relating to diabetes, including β-cell function and glucose levels (supplementary table E12).

## Discussion

This is the first GWAS of PRISm to report genetic associations reaching significance thresholds. We show that there is a heritable component for the development of PRISm. We report 22 distinct signals for PRISm that reach genome-wide significance across both stages of the meta-analysis; all represent novel signals for PRISm. Of these, four SNPs were novel for an association with lung function. This demonstrates the usefulness of performing GWAS of different lung function traits and phenotypes to maximise discovery of heritable genetic variants of lung function and disease.

Genetic correlation and PheWAS studies showed there are shared genetic risk factors with other lung function measures and lung conditions such as COPD (r_g_=0.62) and asthma–COPD overlap (r_g_=0.62), as well as comorbidities of lung disease. In addition to modifiable risk factors such as smoking, COPD can result from a complex interplay of genetic and early life factors that determine lung function trajectories [5, 6]. PRISm is also a heterogeneous state with variable trajectories, of which some progress to COPD over time [2]. Genetic determinants of lung function are associated with COPD [21]. Although not directly tested, given that PRISm and COPD are strongly genetically correlated, this could partially explain the transition between them over time.

PRISm has been consistently associated with systemic comorbidities such as diabetes, heart disease and increased risk of mortality in observational studies [4]. In our analysis, we have shown moderate genetic correlation between PRISm and type 2 diabetes, and the PheWAS showed that 18 of the PRISm SNPs are associated with diabetes, diabetic medication use or hyperglycaemia. PRISm showed positive genetic correlation with type 2 diabetes (r_g_=0.12) and waist–hip ratio (r_g_=0.12) but a weaker and negative genetic correlation with BMI (r_g_= −0.04).

Several observational and cross-sectional studies of impaired lung function (including PRISm) have demonstrated positive associations between PRISm and BMI [7, 22]. Increased BMI has been shown to have stronger associations with restrictive patterns of lung function impairment (such as reduced FVC and FEV_1_, but preserved FEV_1_/FVC ratio, as seen in PRISm) rather than with the classical obstructive pattern [22]. This could be explained by the mechanical effects of adiposity, where fat accumulates around lungs leading to airway narrowing, and around the abdomen impeding chest wall expansion during full inspiration [22, 23]. However, in our analysis, we observed a slight negative correlation between PRISm and BMI. Notably, in the current GWAS, PRISm was adjusted for BMI, which may have affected the direction and magnitude of the genetic correlation observed.

Other proposed mechanisms for the association between obesity and lung function impairment include systemic inflammation, where levels of pro-inflammatory markers such as interleukin-6, C-reactive protein, fibronectin and other cytokines are increased in people with airway obstruction [22, 24]. The relationship between obesity and impaired lung function may also alter with disease progression. While studies have reported positive correlations between PRISm and adiposity, an inverse relationship is commonly reported between adiposity and COPD, with cachexia being a feature of late-stage COPD. Given that genetic correlations alone cannot imply a direction of causation, comparing childhood- and adult-onset obesity as a risk factor for PRISm or COPD may help provide insights into the nature and direction of adiposity effects on lung function phenotypes over the life course. Moreover, given the relationship between height as a precursor of maximally attained lung volume, comparing multiple measures of adiposity (including those that do and do not incorporate height) may inform a deeper understanding of relationships between anthropometric traits and lung function.

In our study, various cardiovascular traits showed a degree of genetic correlation with PRISm. Previous observational and genetic correlation studies have shown associations between cardiovascular traits, including high blood pressure and myocardial infarction, and FEV_1_ and FVC [24, 25]. There are multiple possible mechanisms for this association, including shared risk factors, such as systemic inflammation induced by cigarette smoking; however, a recent study demonstrated that an association between PRISm and cardiovascular disease (CVD) remained after adjustment for multiple risk factors [26]. Other studies have proposed causal mechanisms for a relationship between PRISm and CVD, including a suggestion that congestive heart failure may lead to lung function impairment as a consequence of cardiomegaly and pulmonary congestion [2]. In terms of shared genetic risk factors, beyond shared specific loci for smoking such as the 15q25.1 locus [27], a study performing partitioned genetic correlation analysis demonstrated genetic correlations between COPD and CVD traits, notably in histone markers (h3k9ac, h3k4me3), which play a central role in arterial pressure and bronchial cell development [27, 28]. Genes such as *HHIP* and *EEFSEC* are known to be associated with lung development signalling pathway and translation factors necessary for protein synthesis associated with COPD and cardiovascular events [27].

We found that there is an overlap between the top SNPs in our PRISm GWAS and continuous lung function traits. This is not surprising given PRISm is a diagnosis based on spirometry, as opposed to a disease defined by a unique pathogenic mechanism. Similarly, a previous GWAS of COPD based on spirometric criteria has discovered loci that have been described as associated with a diverse range of lung diseases, including asthma and idiopathic pulmonary fibrosis [29]. This likely reflects the genetic heterogeneity of lung diseases, as well as how spirometric measures are routinely used for the clinical diagnosis and functional assessment of various lung disorders.

It is possible that better sub-phenotyping of PRISm towards a more homogeneous subpopulation (*e.g.* those with PRISm and BMI <25 kg·m^−2^) could lead to the discovery of new genetic associations. Given that a high proportion of those with PRISm transition to other lung function states over time [4], both normal lung function and COPD, then future genetic studies focusing on persistent PRISm or PRISm that progresses to COPD could be informative, although such focus could result in limited sample size and reduced power.

The majority of published GWAS have adopted a significance threshold of p<5×10^−8^ [30], although more liberal and stricter thresholds have been proposed [31]. Specifically, stricter thresholds of 3×10^−8^ [32] or 5×10^−9^ [33] have been proposed for GWAS focusing on low frequency (MAF 1–5%) and rare (MAF <1%) genetic variants, respectively, to correct for increased multiple testing. Had we adopted the stricter definition of p<3×10^−8^, all reported signals would have remained significant. We did not adopt the even stricter threshold of 5×10^−9^ because we excluded rare variants (MAF <1%) from our analysis; however, this may have been at the expense of missing rare variant associations with PRISm.

Population stratification and cryptic relatedness can cause spurious associations in GWAS. We used a linear mixed model for our discovery GWAS, which could account for these issues [34]. To account for genomic inflation we used LD score regression and adjusted for LD intercept. We excluded SNPs that reached significance threshold in Stage 1 analysis if their rsID had been reported as associated with lung function in the largest published lung function GWAS at the time of analysis of Shrine *et al*. [6]. This was in an attempt to focus on SNPs that were novel for PRISm, rather than simple lung function. If they had not been excluded, these SNPs may have been reported in this paper as having an association with PRISm. However, we did not exclude SNPs in LD with previously reported SNPs. Therefore, SNPs in high LD with excluded SNPs may still have been reported. Our GWAS was only performed on those of European heritage; therefore, these results may not be generalisable to other ancestral populations. The SNPs discovered in Stage 1 may not successfully replicate in diverse ancestral populations. The prevalence of PRISm varies by region and ancestral population, from 4.2% in males in Sydney, Australia, to 48.7% in females in Manila, Philippines [35]. Rates of PRISm comorbidities also vary [35]. Future research should aim to recruit from diverse ancestries to explore any heritable component to this variation. Our GWAS was performed using pre-bronchodilator values, although medication was not withheld prior to spirometric testing. Although post-bronchodilator values are not required for PRISm diagnosis, there is evidence that spirometric values can change in those with PRISm post-bronchodilation [36].

### Conclusion

This is the first GWAS of PRISm to successfully identify genetic associations reaching genome-wide significance. We defined 22 genetic signals for PRISm, of which four are also novel for lung function, highlighting that GWAS of different lung function phenotypes are complementary. Genetic risk factors for PRISm overlap with those for other lung diseases and extrapulmonary comorbidities.

## Supplementary material

10.1183/13993003.00337-2023.Supp1 **Please note:** supplementary material is not edited by the Editorial Office, and is uploaded as it has been supplied by the author.
Supplementary material: Deep-PheWAS results ERJ-00337-2023.SUPPLEMENT
Supplementary appendices ERJ-00337-2023.SUPPLEMENT
Supplementary material: PheWAS results ERJ-00337-2023.SUPPLEMENT

## Shareable PDF

10.1183/13993003.00337-2023.Shareable1 This one-page PDF can be shared freely online.
Shareable PDF ERJ-00337-2023.Shareable
