# Supplementary material for: Genome-wide association study of preserved ratio impaired spirometry (PRISm)
Source: Eur Respir J. 2024 Jan 4;63(1):2300337. doi: 10.1183/13993003.00337-2023 (PMC10765494; doi:10.1183/13993003.00337-2023)
Supplement: Supplementary file 2 [file ERJ-00337-2023.SUPPLEMENT.pdf]

Supplementary Information

Contents

Appendix 1. Spirometry methods
Appendix 2. QQ plot
Appendix 2. LD correlation analysis – full results and GWAS used
Appendix 3. Description of replicating cohorts
Appendix 4. Replicating cohorts' analysis methods
Appendix 5. Deep-PheWAS graphs
Appendix 6. Strongest r^2 for SNPs novel for lung function
Appendix 7. Gene expression heat map of nearest gene to 4 SNPs novel for lung function
Appendix 8. Results of FUMA Gene-set enrichment analysis for 26 nearest genes from Table 2

Appendix 1.

All participants were requested to perform pre-bronchodilator spirometry, using Vitalograph Pneumotrac 6800. The participants were asked to record two to three blows (lasting for at least 6 seconds) within a period of about 6 minutes. The computer compared the reproducibility of the first two blows and, if acceptable (defined as a <5% difference in FVC and FEV_1), indicated that the third blow was not required. The highest measures of FEV_1 and FVC from acceptable blows was used.

Appendix 2. QQ plot

Figure E1. Quartile-Quartile Plot of discovery GWAS

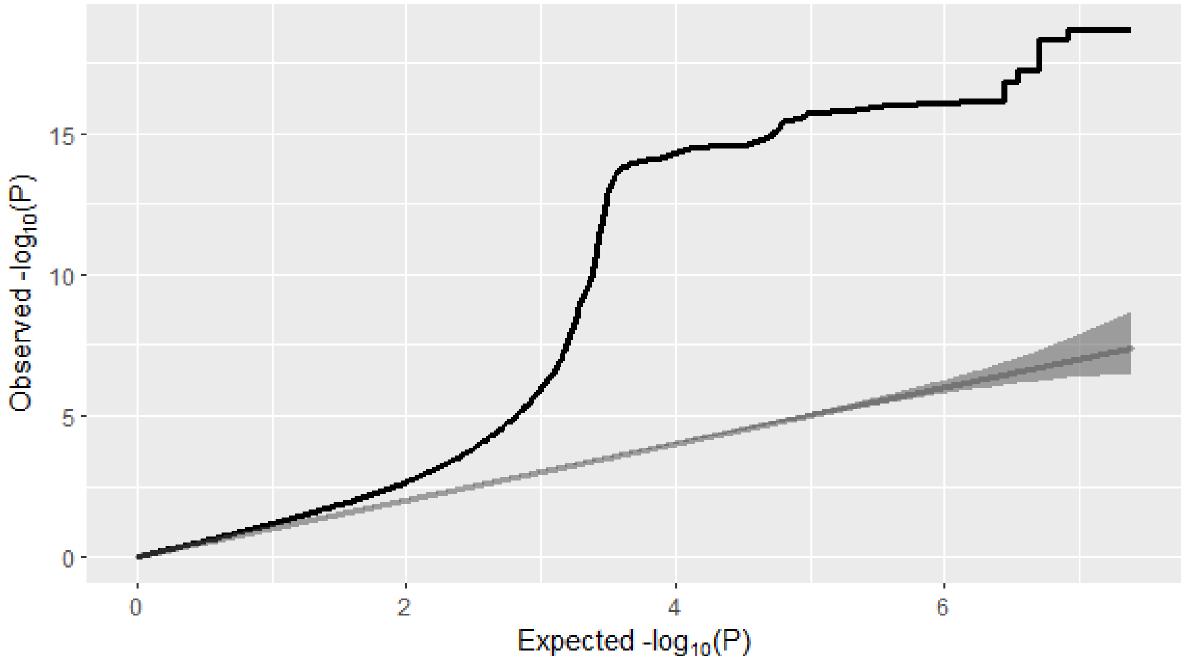

As mentioned in the discussion the QQ plot shows a large increase in observed hits compared to expected hits. This is likely due to the large number of reported SNPs reaching p-value $<5^{-8}$ (N =6,037). However, after clumping there were 26 SNPs considered for further analysis.

2. LD-correlation analysis

Quantitative lung function GWASs

We used published summary-level data from three GWAS of FEV1, FVC and FEV1/FVC, undertaken in UK Biobank (N=321,047) and the SpiroMeta consortium (N=79,055). Prior to GWAS, traits were pre-adjusted for age, age2, sex, height, smoking status and other covariates as appropriate, e.g. ancestry principal components. Residuals were inverse-normal rank transformed.[1,2]

Table E1. LD correlation of UK Biobank PRISm GWAS and UK Biobank+SpiroMeta consortium lung function GWAS

Lung function GWAS	rg	se	z	p	h2_obs	h2_obs_se	h2_int	h2_int_se	gcov_int	gcov_int_se
FEV1	-0.956	0.0118	-81.0769	0.0	0.064	0.0032	1.0194	0.0083	-0.407	0.0083
FVC	-0.9282	0.0118	-78.4733	0.0	0.064	0.0032	1.0194	0.0082	-0.4109	0.0081
PEF	-0.6502	0.0192	-33.8372	5.6060e-251	0.064	0.0032	1.0195	0.0083	-0.2746	0.0071
RATIO	-0.2192	0.0264	-8.3069	9.8248e-17	0.064	0.0032	1.0194	0.0082	-0.0581	0.0077

Moderate-to-severe asthma

We used a published GWAS of moderate-to-severe asthma within the Genetics of Asthma Severity and Phenotypes (GASP) initiative, with additional cases included from the U-BIOPRED asthma cohort, and UK Biobank. All cases (N=5135) were taking medication for asthma, and met the criteria for moderate-to-severe asthma according to the British Thoracic Society (BTS) 2014 guidelines. Controls (N=25,675) were from UK Biobank, and excluded those with a doctor-diagnosis of asthma, rhinitis, eczema, allergy, emphysema, or chronic bronchitis, or those with missing medication data. Analyses were adjusted for the first 10 principal components.[1,3]

Table E2. LD correlation of UK Biobank PRISm GWAS and Moderate-to-severe asthma GWAS

	rg	se	z	p	h2_obs	h2_obs_se	h2_int	h2_int_se	gcov_int	gcov_int_se
Mod-severe asthma	0.3099	0.0503	6.1584	7.3472e-10	0.0633	0.0032	1.0203	0.0101	0.0083	0.0063

Asthma-COPD overlap (ACO)

We defined ACO in UK Biobank (N=8,068) as individuals self-reporting a doctor diagnosis of asthma, and with FEV1/FVC<0.7 and FEV1<80% predicted at any study visit. Controls (N=40,360) were selected in approximately a 5:1 ratio, from participants reporting no asthma or COPD, (FEV1 >80% predicted, and FEV1/FVC>0.7). Associations were adjusted for age (at recruitment), sex, smoking status, and 10 ancestry principal components.[1,4]

Table E3. LD correlation of UK Biobank PRISm GWAS and Asthma-COPD overlap (ACO) GWAS

	rg	se	z	p	h2_obs	h2_obs_se	h2_int	h2_int_se	gcov_int	gcov_int_se
ACOS	0.5174	0.0385	13.451	3.0389e-41	0.064	0.0032	1.0193	0.0083	0.0585	0.0057

Respiratory infections

We defined respiratory tract infections requiring hospital admission in UK Biobank, using a range of ICD-10 codes. Cases had one or more admission for respiratory infections (N=19,459). Controls had no admissions for respiratory infections and were selected in approximately a 5:1 ratio (N=101,438). Associations were adjusted for age (at recruitment), age2, sex, smoking status, genotyping array, and 10 ancestry principal components.[1]

Table E4. LD correlation of UK Biobank PRISm GWAS and Respiratory Infections GWAS

	rg	se	z	p	h2_obs	h2_obs_se	h2_int	h2_int_se	gcov_int	gcov_int_se
Respiratory Infections	0.1849	0.0626	2.9524	0.0032	0.064	0.0032	1.0195	0.0082	0.0285	0.0052

Eosinophils

We used a published GWAS of blood eosinophils in UK Biobank/INTERVAL studies. Prior to GWAS, eosinophil counts were adjusted for biological and technical laboratory covariates, and GWAS results were provided as SD change in transformed cell count, per risk allele. Adjustments were made for technical and seasonal covariates, as well as age, menopausal status, height, weight, smoking and alcohol.[1,5]

Table E5. LD correlation of UK Biobank PRISm GWAS and Eosinophil GWAS

	rg	se	z	p	h2_obs	h2_obs_se	h2_int	h2_int_se	gcov_int	gcov_int_se
Eosinophils	0.0618	0.0246	2.5077	0.0122	0.0641	0.0032	1.0192	0.0082	0.0166	0.0058

Table E6. Body mass index[6]

	rg	se	z	p	h2_obs	h2_obs_se	h2_int	h2_int_se	gcov_int	gcov_int_se
BMI	-0.0383	0.0178	-2.1552	0.0311	0.064	0.0031	1.0159	0.0084	-0.02	0.0086

Table E7. Hypertension[7]

	rg	se	z	p	h2_obs	h2_obs_se	h2_int	h2_int_se	gcov_int	gcov_int_se
HTN_SBP	0.0849	0.0226	3.7638	0.0002	0.0646	0.0033	1.0165	0.009	0.0113	0.0086
HTN_DBP	0.0497	0.0235	2.1129	0.0346	0.0645	0.0032	1.017	0.0089	0.0019	0.009

Table E8. Myocardial Infarction[8]

	rg	se	z	p	h2_obs	h2_obs_se	h2_int	h2_int_se	gcov_int	gcov_int_se
MI	0.0693	0.0259	2.6783	0.0074	0.0641	0.0032	1.0184	0.0083	0.012	0.0051

Table E9. Type 2 Diabetes Mellitus[9]

	rg	se	z	p	h2_obs	h2_obs_se	h2_int	h2_int_se	gcov_int	gcov_int_se
T2D	0.1224	0.0298	4.1065	4.0166e-05	0.0656	0.0033	1.0035	0.0112	0.0327	0.0087

3. Description of replicating cohorts

Agricultural Lung Health Study (ALHS)
The ALHS is a case–control study of current asthma among farmers and their spouses nested within the prospective Agricultural Health Study. Detailed descriptions are provided at the references.[10] Lung function was measured by trained staff during in-home visits using an EasyOne Spirometer (NDD Medical Technologies, Chelmsford, Massachusetts, USA) based on the American Thoracic Society (ATS) guidelines.

Cardiovascular Health Study (CHS)
CHS recruited 5,888 men and women aged 65 or older in four U.S. communities—Sacramento, CA; Hagerstown, MD; Winston-Salem, NC; and Pittsburgh, PA—conducting annual clinical exams between 1989 and 1999. A standard spirometry system, validated by a third party to meet ATS spirometer recommendations, was used by all four clinical centres. A water-sealed spirometer connected to a personal computer was used. Software assisted the technician with quality control (QC) of manoeuvres, calculated the lung function variables. Full details of cohort and recruitment are described at the references.[11-13] For details on lung function measures see reference.[14]

The Genetic Epidemiology of COPD (COPDGene)
The Genetic Epidemiology of COPD (COPDGene. ClinicalTrials.gov Identifier NCT000608764) study is a noninterventional, multicentre, longitudinal analysis of > 10,000 subjects, including smokers with a ≥ 10 pack-year history with and without COPD and healthy never smokers. The details of COPDGene recruitment has been described previously at the reference.[15]

Danish study of Functional Disorders (DanfunD)
Danish study of Functional Disorders (DanFunD) cohort was established to outline the epidemiology of functional somatic syndromes and is described in detail at the referece.[16] The study population comprises a random sample of 9,656 men and women aged 18-76 years from the general population examined from 2011 to 2015.

Extended Cohort for E-health, Environment and DNA
The Extended Cohort for E-health, Environment and DNA (EXCEED) is an on-going longitudinal population-based cohort in Leicester, Leicestershire, and Rutland including diverse population from Black, Asian, and minority ethnic (BAME) communities. EXCEED aims to understand the determinants of diseases and multi-morbidity in the context of genetics, environmental, and lifestyle using baseline data on demographics, anthropometry, spirometry, lifestyle factors and electronic health care records linkage. Over 10,000 participants have been enrolled since the beginning of 2013.[17]

In this study, 3441 participants that had spirometry measurements and demographic data were screened. All implausible measures including extreme of age and height, sex mismatches, lacking date of birth, sex and height records, and encoding errors were identified and corrected by comparing the study records and electronic health care records. After merging this with genetic data and passing the quality control, 1480 participants remained for analysis. Further quality control measures were done. Absolute spirometry values were converted to Z-scores using the GLI-2012 equations in the 'rspiro' R package and demographic data (e.g. age, height, gender, and ethnicity). No individual had an absolute Z-score value of greater than 5 (the criterion for outlier removal).

Framingham Heart Study
The Framingham Heart Study (FHS) has collected spirometry and smoking history data on three generations of adults, with recruitment of the original cohort beginning in 1948. Full recruitment details are provided elsewhere.[18] Participants provided DNA samples that have been genotyped using microarray technology. These genotype and phenotype data, which have been made publicly

available through the NHLBI's SNP Health Association Resource (SHARe) initiative. (http://public.nhlbi.nih.gov/GeneticsGenomics/home/share.aspx),

Health2006
The Health2006 cohort is described in detail elsewhere.[19] Recruitment began in 2006 to investigate lifestyle related chronic health conditions. Participants were drawn as a randomn sample from the south-western part of the Great Copehagen area between the ages of 18-69. 44.7% of the 2471 recruited participated in a health examination between June 2006 and June 2008, with ongoing follow up at five-year time points. Participants were recruited and examined in the same manner for the Health2008 and Health2010 cohorts which supply additional corresponding data. Spirometry was measured using the SpiroUSB (MicroMedical Ltd, Rochester, UK) in accordance with ERS standards. Details about spirometric measures can be found at the reference.[20] Fasting venous blood was taken from all participants for Biobank purposes. DNA was extracted using a Qiagen AutoPure LS system.

Inter99
The Inter99 study comprises an age- and sex-stratified sample of 13016 participants residing in 11 suburban municipalities of the south-western part of Copenhagen County. The study sample was randomized from the Danish Civil Registration System that comprises all participants permanently residing in Denmark. A total of 6784 participants aged 30–60 years underwent the screening-program at the Research Centre for Prevention and Health at Copenhagen University Hospital in Glostrup, Denmark.[21]

Lifelines
The Lifelines Cohort Study has been described in detail elsewhere.[22] It is a large population-based cohort study that recruited participants between 2006 and 2013 from inhabitants of the northern part of The Netherlands. Family members were recruited leading to a three-generation design. Baseline data was collected from 167729 participants aged 6months to 93years. Follow-up visits occur every 5 years with data linkage to medical registries and environmental data. Pre-bronchodilator spirometry was performed with a Welch Allyn Version 1.6.0.489, PC-based Spiroperfect with CA Workstation software according to ATS/ERS guidelines. Technical quality and results were evaluated by well-trained assistants and difficult to interpret results were re-evaluated by a lung physician.[23]

Lung Health Study
Lung Health Study (LHS) was a multicentre study evaluating the effects of ipratropium bromide and smoking cessation on lung function decline (FEV_1) in smokers with mild to moderate COPD. Full description has been done elsewhere.[24,25] Individuals with clinical COPD at the beginning of the study were not eligible for the LHS. This study was carried-out over five years. LHS included healthy smokers aged 35-60 years at risk of COPD based on mild airway obstruction. Spirometry was taken at the baseline visit and once a year at each follow-up visit.

Lothian Birth Cohort
Details of the Lothian Birth Cohort 1936 have been previously published.[26] Lung function assessing forced expiratory volume in 1 s and forced vital capacity (each the best of three), using a Micro Medical Spirometer was assessed sitting down without nose clips at age ~70 years. The accuracy of the spirometer is ±3% (to ATS recommendations Standardisation of Spirometry 1994 update for flows and volumes).

Multi-Ethnic Study of Atherosclerosis

The Multi-Ethnic Study of Atherosclerosis (MESA) recruitment process has been described in detail elsewhere.[27] In brief MESA is a prospective community-based study that has recruited 6814 non-Hispanic white, black, Hispanic, and Chinese American participants, aged 45 through 84 years in the years 2000 through 2002 from the general population in 6 US communities. In a sub-study, MESA Lung, participants had performed full-lung CT and spirometry in years 2010 through 2012, with a follow-up assessment in years 2016 through 2018.[28] Spirometry was performed at baseline using ATS recommendations.

Rotterdam Study

The Rotterdam study is a prospective cohort study ongoing since 1990 in the city of Rotterdam in the Netherlands. As of 2008 about 15,000 subjects aged 45 years or over comprise the Rotterdam Study cohort. For full details see the reference.[29] Pre-bronchodilator spirometry was performed during the research centre visit using a SpiroPro portable spirometer (RS-III-1) or a Master Screen® PFT Pro (RS-BIOS) by trained para-medical staff according to the ERS/ATS Guidelines.[23]

4. Replicating cohorts' analysis methods

Table E10. Stage 2 studies analysis methods

Study	Individual call rate filter	SNP call rate filter	Other filters	GWAS platform	Imputation panel	Imputation server	N case:control
ALHS	0.95	0.95	failed Affy QC, failed plate effect check	UK Biobank Axiom Array	HRC 1.1	Michigan	342:1722
CHS	0.95	0.97	genotype discordant with known sex or prior genotyping	Illumina 370CNV BeadChip System	HRC 1.1	Michigan	208:1476
COPDGene	0.98	0.98	-	PLINK 2.0 alpha	HRC 1.1	Michigan	698:2534
DanFunD	0.98	0.98	-	SNPtest 2.5.2	HRC	Michigan	423:4782
EXCEED	0.95	0.95	-	UK Biobank Axiom Array	HRC 1.1	Michigan	256:803
FHS	0.97	0.97	At locations that did not map to GRCh37	Affymetrix 500K mapping array plus Affymetrix 50K supplemental array	HRC 1.1	Michigan	265:4830
Health	0.98	0.98	-	SNPtest 2.5.2	HRC	Michigan	448:3619
Inter99	0.98	0.98	-	SNPtest 2.5.2	HRC	Michigan	577:3930
Lifelines	0.99	0.99	-	Infinium Global Screening Array®(GSA) MultiEthnic Disease Version 1.0	HRC 1.1	Sanger	968:18483
LHS	0.999	0.85	missingness > 2%, PCA outliers	Illumina Human660W-Quad v.1_A BeadChip	HRC 1.1	Michigan	264:617
Lothian	0.95	0.98	discrepancy between genetic and reported sex	Illumina Human610-Quadv1	HRC 1.1	Michigan	275:555

MESA	0.95	0.95	1) variants that don't match on chromosome, position, and allele; 2) variants not present in the HRC reference; 3) all AT/GC SNPs with MAF >40% in the reference data set; 4) all SNPs with an allele frequency difference >0.2, between reference and data set frequency file; 5) duplicates that may be introduced with the chromosome/position update.	Affymetrix Human SNP array 6.0	HRC 1.1	Michigan	128:923
Rotterdam	0.98	0.95	(1) individuals with low genotype completion rate (<90%), (2) individuals with sex-mismatches, (3) one individual from duplicate pairs. Variants not called in over 5% of the individuals and those that deviated significantly form the expected Hardy–Weinberg Equilibrium proportions (P < 1 × 10−06).	Illumina 550L, 550K duo or 610 quad arrays	HRC 1.1	haplotype reference consortium	313:3509

<u>Appendix 5. Deep-PheWAS figures</u>

Figure E2. Deep-PheWAS result of rs1233604

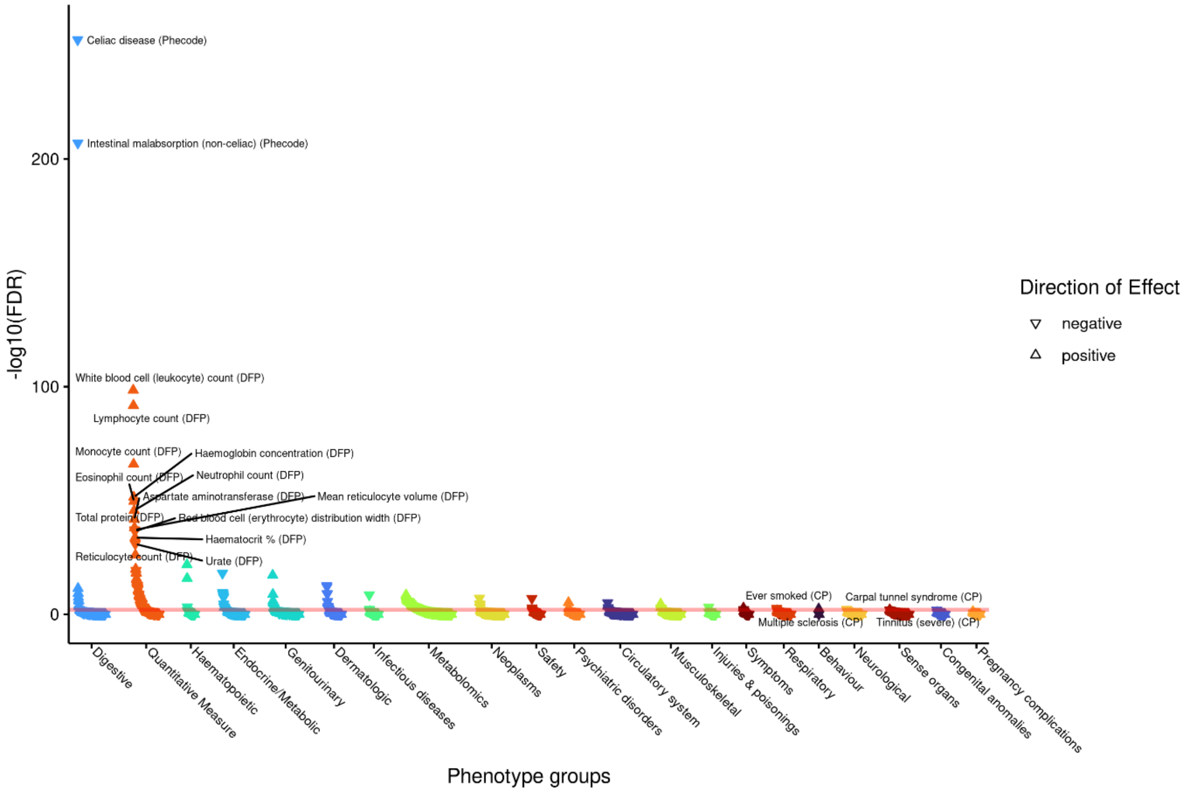

Figure E3. Deep PheWAS result of rs7652391

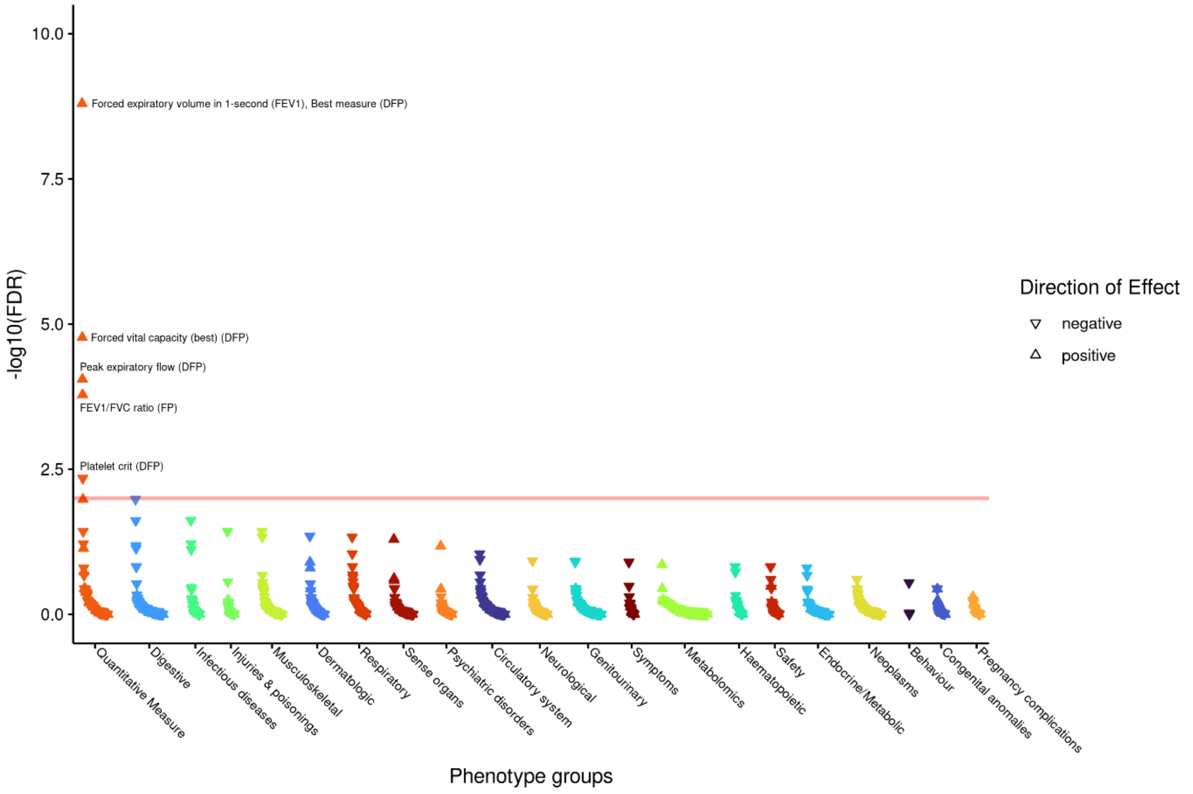

Figure E4. Deep PheWAS result of rs9431040

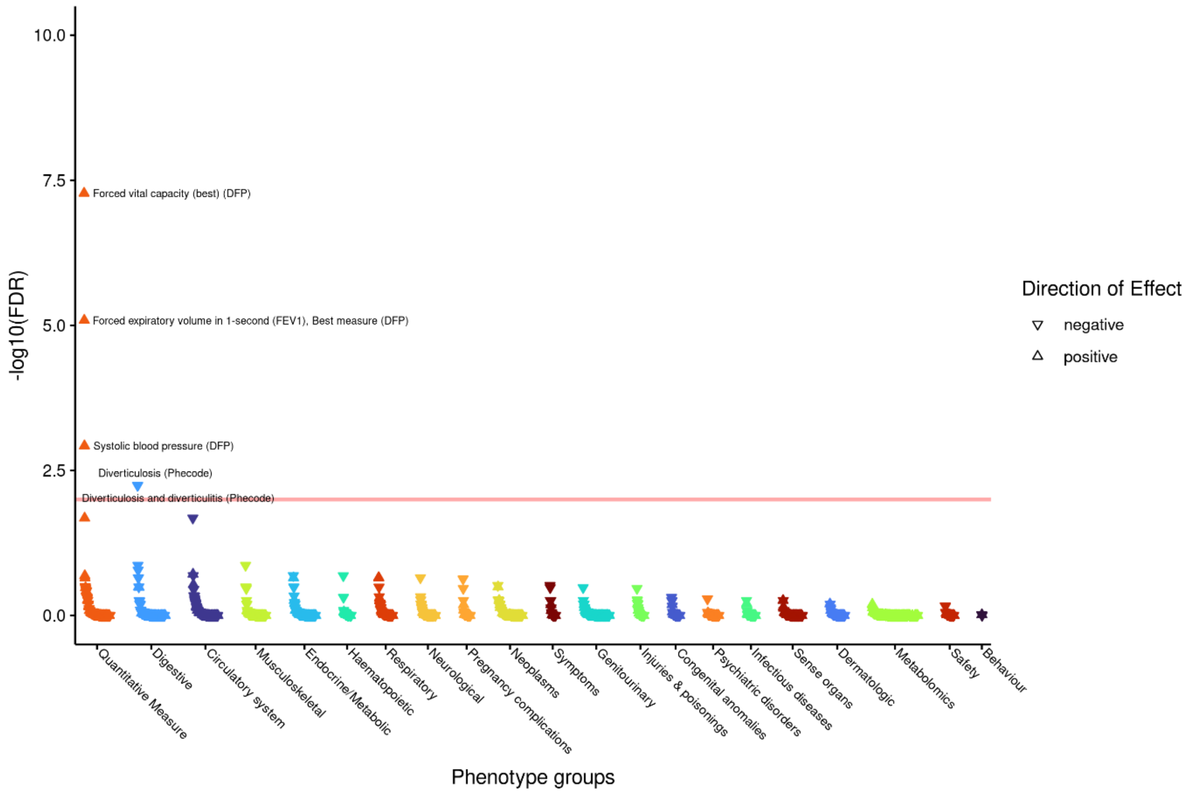

Figure E5. Deep PheWAS result of rs62018863

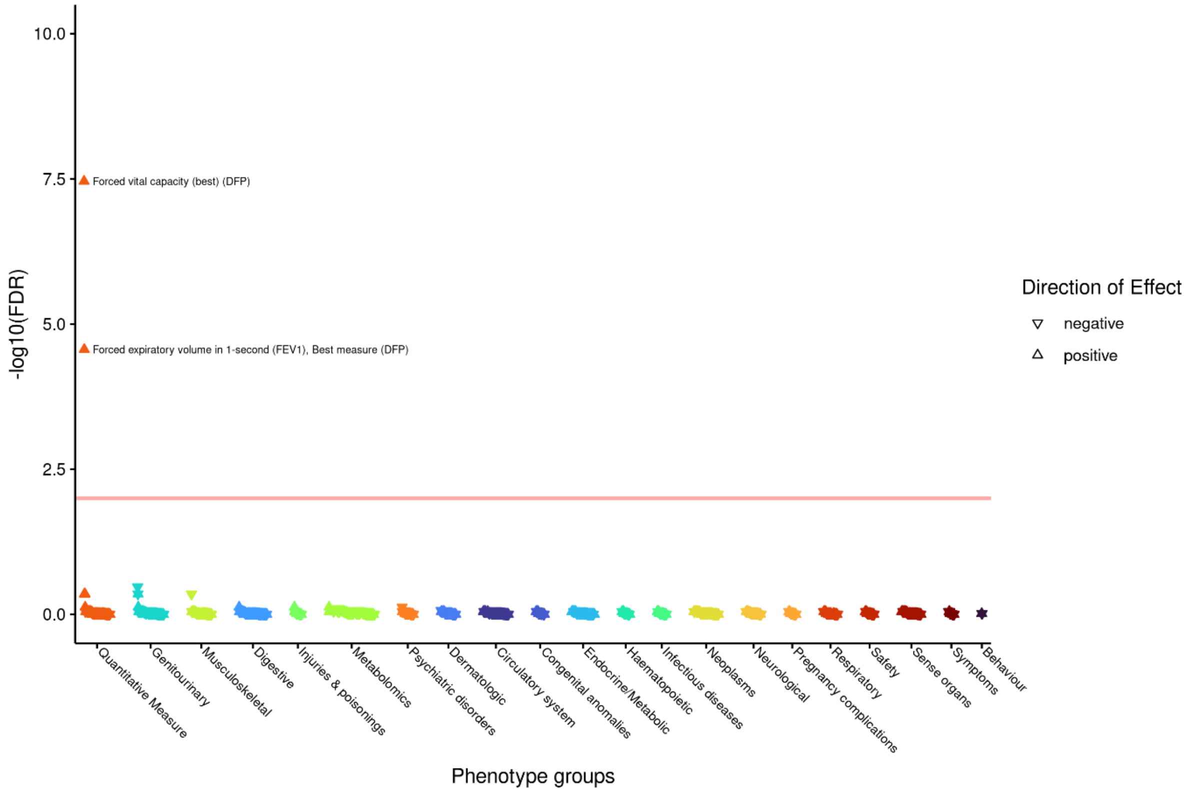

Figure E5. Deep PheWAS result of rs185937162

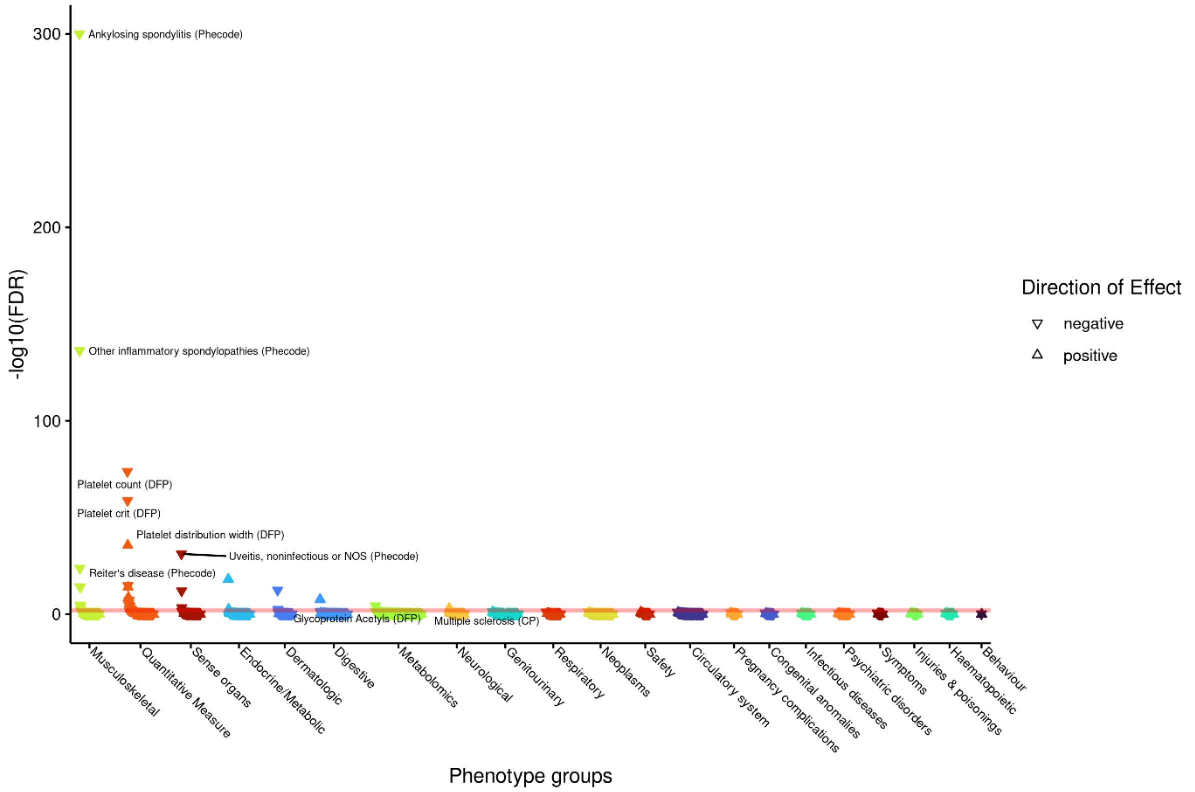

Appendix 6.

Table E11. Strongest LD of four PRISm signals not found in Shrine et al 2022 GWAS

CHR	PRISM	POS	LF	POS	R^2
1	rs9431040	221,152,299	rs11118652	221,270,457	0.161
3	rs7652391	168,913,273	rs879394	168,709,843	0.338
6	rs185937162	31,325,268	rs58490916	31,270,386	0.441
16	rs62018863	8,624,118	rs7198383	8,619,750	0.274

CHR – chromosome. PRISM – rsid in PRISM GWAS. LF – rsid of SNP with largest r2 in Shrine et all 2022 GWAS. POS – position. R^2 between PRISm and LF SNP

Appendix 7.

Figure E6. Gene expression heat map of nearest gene to 4 SNPs novel for lung function

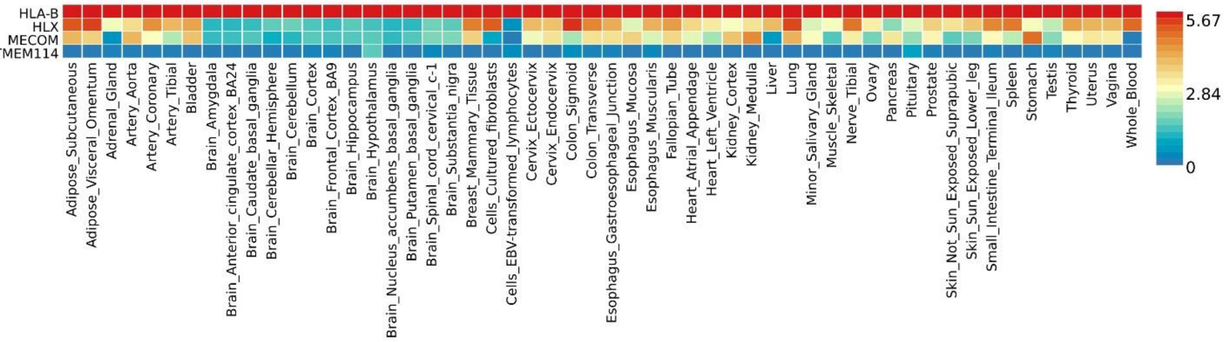

Gene expression figure created by FUMA using GTEx v8 54 tissue types. Expression value is average expression per label (log2 transformed)[30]

Table E12. Results of FUMA Gene-set enrichment analysis for 26 nearest genes from Table 2

GeneSet	N_genes	N_overlap	P	adjP	*Genes*
Lung function (FVC)	143	6	2.06x10-9	5.84x10-6	*HLX, MECOM, BMP6, GLIS3, EML3, RIN3*
FEV_1	149	6	2.64x10-9	5.84x10-6	*HLX, MECOM, BMP6, GLIS3, EML3, RIN3*
White blood cell count	467	6	2.28x10-6	0.003	*HLX, MECOM, BMP6, HLA-B, GLIS3, RIN3*
Type 2 diabetes	541	6	5.33x10-6	0.006	*HLA-B, DGKB, GLIS3, ZMIZ1, SLX4, TMEM114*
Chronic obstructive pulmonary disease	146	4	7.79x10-6	0.007	*MECOM, BMP6, GLIS3, RIN3*
Neutrophil count	361	5	1.32x10-5	0.009	*HLX, HLA-B, ZMIZ1, RIN3, ZGPAT*
Waist circumference adjusted for body mass index	638	6	1.37x10-5	0.009	*HLX, BMP6, HLA-B, DGKB, ZMIZ1, EML3*
Monocyte count	380	5	1.69x10-5	0.009	*MECOM, HLA-B, GLIS3, ZMIZ1, RIN3*
Composite immunoglobulin trait (IgA/IgM)	8	2	2.16x10-5	0.010	*HLA-B, RIN3*
Lung function (FEV_1)	66	3	2.74x10-5	0.010	*GLIS3, RIN3, ZGPAT*
Homeostasis model assessment of beta-cell function	9	2	2.78x10-5	0.010	*DGKB, GLIS3*
White matter integrity (mean diffusivity)	9	2	2.78x10-5	0.010	*ZFP57, HLA-B*
Takayasu arteritis	70	3	3.27x10-5	0.011	*HLA-B, ZMIZ1, TMEM114*
Hip circumference adjusted for BMI	820	6	5.65x10-5	0.018	*MECOM, BMP6, ZFP57, HLA-B, ZMIZ1, EML3*
Nasopharyngeal carcinoma	13	2	6.01x10-5	0.018	*MECOM, HLA-B*
Platelet count	557	5	1.05x10-4	0.028	*MECOM, BMP6, HLA-B, GLIS3, ZGPAT*
Neutrophil percentage of white cells	286	4	1.08x10-4	0.028	*HLA-B, ZMIZ1, RIN3, ZGPAT*
Blood sugar levels	19	2	1.31x10-4	0.032	*DGKB, GLIS3*
Lymphocyte percentage of white cells	305	4	1.39x10-4	0.032	*GLIS3, ZMIZ1, RIN3, ZGPAT*
Appendicular lean mass	603	5	1.53x10-4	0.034	*MECOM, BMP6, HLA-B, GLIS3, RIN3*
Monocyte percentage of white cells	334	4	1.97x10-4	0.041	*HLX, MECOM, HLA-B, ZMIZ1*

Gene-Set Enrichment Analysis was run using FUMA, setting the 'background genes' list to 20,260 protein coding genes (The full list of gene-sets included in FUMA is given at: https://fuma.ctglab.nl/tutorial#gene2func). N_genes = number of genes in pathway; N_overlap = number of overlapping genes with 26 input genes from Table 2. P and adjP = P-value for hypergeometric test, and same p-value adjusted for multiple test correction, performed per category (i.e. separately for canonical pathways, GO biological processes, etc.).

1. Guyatt AL, John C, Williams AT, et al. Mendelian randomisation analyses of eosinophils and other blood cell types in relation to lung function and disease. *medRxiv* 2020: 2020.07.09.20148726.
2. Shrine N, Guyatt AL, Erzurumluoglu AM, et al. New genetic signals for lung function highlight pathways and chronic obstructive pulmonary disease associations across multiple ancestries. *Nature Genetics* 2019; **51**(3): 481-93.
3. Shrine N, Portelli MA, John C, et al. Moderate-to-severe asthma in individuals of European ancestry: a genome-wide association study. *The Lancet Respiratory medicine* 2019; **7**(1): 20-34.
4. John C, Guyatt AL, Shrine N, et al. Genetic associations and architecture of asthma-chronic obstructive pulmonary disease overlap. *medRxiv* 2020: 2020.11.26.20236760.
5. Astle WJ, Elding H, Jiang T, et al. The Allelic Landscape of Human Blood Cell Trait Variation and Links to Common Complex Disease. *Cell* 2016; **167**(5): 1415-29.e19.
6. Yengo L, Sidorenko J, Kemper KE, et al. Meta-analysis of genome-wide association studies for height and body mass index in ~700000 individuals of European ancestry. *Hum Mol Genet* 2018; **27**(20): 3641-9.
7. Evangelou E, Warren HR, Mosen-Ansorena D, et al. Genetic analysis of over 1 million people identifies 535 new loci associated with blood pressure traits. *Nature Genetics* 2018; **50**(10): 1412-25.
8. Hartiala JA, Han Y, Jia Q, et al. Genome-wide analysis identifies novel susceptibility loci for myocardial infarction. *European Heart Journal* 2021; **42**(9): 919-33.
9. Xue A, Wu Y, Zhu Z, et al. Genome-wide association analyses identify 143 risk variants and putative regulatory mechanisms for type 2 diabetes. *Nature Communications* 2018; **9**(1): 2941.
10. House JS, Wyss AB, Hoppin JA, et al. Early-life farm exposures and adult asthma and atopy in the Agricultural Lung Health Study. *The Journal of allergy and clinical immunology* 2017; **140**(1): 249-56.e14.
11. https://chs-nhlbi.org/. 2021.
12. Fried LP, Borhani NO, Enright P, et al. The Cardiovascular Health Study: design and rationale. *Ann Epidemiol* 1991; **1**(3): 263-76.
13. Tell GS, Fried LP, Hermanson B, Manolio TA, Newman AB, Borhani NO. Recruitment of adults 65 years and older as participants in the Cardiovascular Health Study. *Ann Epidemiol* 1993; **3**(4): 358-66.
14. Griffith KA, Sherrill DL, Siegel EM, Manolio TA, Bonekat HW, Enright PL. Predictors of Loss of Lung Function in the Elderly. *American journal of respiratory and critical care medicine* 2001; **163**(1): 61-8.
15. Regan EA, Hokanson JE, Murphy JR, et al. Genetic epidemiology of COPD (COPDGene) study design. *Copd* 2010; **7**(1): 32-43.
16. Dantoft TM, Ebstrup JF, Linneberg A, et al. Cohort description: The Danish study of Functional Disorders. *Clin Epidemiol* 2017; **9**: 127-39.
17. John C, Reeve NF, Free RC, et al. Cohort Profile: Extended Cohort for E-health, Environment and DNA (EXCEED). *International journal of epidemiology* 2019; **48**(3): 678-9j.
18. Splansky GL, Corey D, Yang Q, et al. The Third Generation Cohort of the National Heart, Lung, and Blood Institute's Framingham Heart Study: design, recruitment, and initial examination. *Am J Epidemiol* 2007; **165**(11): 1328-35.
19. Thuesen BH, Cerqueira C, Aadahl M, et al. Cohort Profile: the Health2006 cohort, research centre for prevention and health. *International journal of epidemiology* 2014; **43**(2): 568-75.
20. Hersoug LG, Husemoen LL, Thomsen SF, Sigsgaard T, Thuesen BH, Linneberg A. Association of indoor air pollution with rhinitis symptoms, atopy and nitric oxide levels in exhaled air. *International archives of allergy and immunology* 2010; **153**(4): 403-12.
21. Drobnjak D, Munch IC, Glümer C, et al. Relationship between retinal vessel diameters and retinopathy in the Inter99 Eye Study. *Journal of Clinical & Translational Endocrinology* 2017; **8**: 22-8.

22. Scholtens S, Smidt N, Swertz MA, et al. Cohort Profile: LifeLines, a three-generation cohort study and biobank. *International journal of epidemiology* 2014; **44**(4): 1172-80.
23. de Vries M, Nedeljkovic I, van der Plaat DA, et al. DNA methylation is associated with lung function in never smokers. *Respiratory research* 2019; **20**(1): 268.
24. Hansel NN, Ruczinski I, Rafaels N, et al. Genome-wide study identifies two loci associated with lung function decline in mild to moderate COPD. *Hum Genet* 2013; **132**(1): 79-90.
25. Anthonisen NR, Connett JE, Kiley JP, et al. Effects of smoking intervention and the use of an inhaled anticholinergic bronchodilator on the rate of decline of FEV1. The Lung Health Study. *Jama* 1994; **272**(19): 1497-505.
26. Taylor AM, Pattie A, Deary IJ. Cohort Profile Update: The Lothian Birth Cohorts of 1921 and 1936. *International journal of epidemiology* 2018; **47**(4): 1042-r.
27. Bild DE, Bluemke DA, Burke GL, et al. Multi-Ethnic Study of Atherosclerosis: objectives and design. *Am J Epidemiol* 2002; **156**(9): 871-81.
28. Smith BM, Kirby M, Hoffman EA, et al. Association of Dysanapsis With Chronic Obstructive Pulmonary Disease Among Older Adults. *Jama* 2020; **323**(22): 2268-80.
29. Hofman A, Breteler MM, van Duijn CM, et al. The Rotterdam Study: objectives and design update. *Eur J Epidemiol* 2007; **22**(11): 819-29.
30. Watanabe K, Taskesen E, van Bochoven A, Posthuma D. Functional mapping and annotation of genetic associations with FUMA. *Nature communications* 2017; **8**(1): 1826.
