# Supplementary material for: Genome-wide association study of preserved ratio impaired spirometry (PRISm)
Source: Eur Respir J. 2024 Jan 4;63(1):2300337. doi: 10.1183/13993003.00337-2023 (PMC10765494; doi:10.1183/13993003.00337-2023)
Supplement: Supplementary file 4 [file ERJ-00337-2023.Shareable.pdf]

EUROPEAN RESPIRATORY JOURNAL
ORIGINAL RESEARCH ARTICLE
D.H. HIGBEE ET AL.

SHAREABLE PDF

Genome-wide association study of preserved ratio impaired spirometry (PRISm)

Daniel H. Higbee[1,2], Alvin Lirio[3], Fergus Hamilton[1], Raquel Granell[1], Annah B. Wyss[4], Stephanie J. London[4], Traci M. Bartz[5], Sina A. Gharib[6], Michael H. Cho[7,8], Emily Wan[7,8,9], Edwin Silverman[9], James D. Crapo[10], Jesus V.T. Lominchar[11], Torben Hansen[11], Niels Grarup[11], Thomas Dantoft[12], Line Kårhus[12], Allan Linneberg[12], George T. O'Connor[13,14], Josée Dupuis[15], Hanfie Xu[16], Maaike M. De Vries[17,18], Xiaowei Hu[19], Stephen S. Rich[19], R. Graham Barr[20], Ani Manichaikul[19], Sara R.A. Wijnant[21,22,23], Guy G. Brusselle[23,24], Lies Lahousse[21,22], Xuan Li[25], Ana I. Hernández Cordero[25], Ma'en Obeidat[25], Don D. Sin[25,26], Sarah E. Harris[27], Paul Redmond[27], Adele M. Taylor[27], Simon R. Cox[27], Alexander T. Williams[3], Nick Shrine[3], Catherine John[3], Anna L. Guyatt[3], Ian P. Hall[28], George Davey Smith[1], Martin D. Tobin[3,29,30] and James W. Dodd[1,2,30]

[1]MRC Integrative Epidemiology Unit (IEU), University of Bristol, Bristol, UK. [2]Academic Respiratory Unit, University of Bristol, Southmead Hospital, Bristol, UK. [3]Department of Population Health Sciences, University of Leicester, Leicester, UK. [4]Epidemiology Branch, National Institute of Environmental Health Sciences, National Institutes of Health, Research Triangle Park, NC, USA. [5]Cardiovascular Health Research Unit, Departments of Biostatistics and Medicine, University of Washington, Seattle, WA, USA. [6]Computational Medicine Core, Center for Lung Biology, UW Medicine Sleep Center, Department of Medicine, University of Washington, Seattle, WA, USA. [7]Channing Division of Network Medicine, Brigham and Women's Hospital, Boston, MA, USA. [8]Harvard Medical School, Boston, MA, USA. [9]Pulmonary and Critical Care Section, Department of Medicine, VA Boston Healthcare System, Boston, MA, USA. [10]Division of Pulmonary, Critical Care and Sleep Medicine, National Jewish Health, Denver, CO, USA. [11]Novo Nordisk Foundation Center for Basic Metabolic Research, Faculty of Health and Medical Sciences, University of Copenhagen, Copenhagen, Denmark. [12]Center for Clinical Research and Prevention, Bispebjerg and Frederiksberg Hospital, Frederiksberg, Denmark. [13]Pulmonary Center, School of Medicine, Boston University, Boston, MA, USA. [14]Division of Pulmonary, Allergy, Sleep, and Critical Care Medicine, Boston Medical Center, Boston, MA, USA. [15]Department of Epidemiology, Biostatistics and Occupational Health, McGill University, Montreal, QC, Canada. [16]Department of Biostatistics, Boston University School of Public Health, Boston, MA, USA. [17]Department of Epidemiology, University of Groningen, University Medical Center Groningen, Groningen, The Netherlands. [18]Groningen Research Institute for Asthma and COPD, University of Groningen, University Medical Center Groningen, Groningen, The Netherlands. [19]Center for Public Health Genomics, University of Virginia, Charlottesville, VA, USA. [20]Department of Medicine, College of Physicians and Surgeons, Columbia University, New York, NY, USA. [21]Department of Bioanalysis, Ghent University, Ghent, Belgium. [22]Department of Epidemiology, Erasmus MC, Rotterdam, The Netherlands. [23]Department of Respiratory Medicine, Ghent University Hospital, Ghent, Belgium. [24]Department of Epidemiology, Department of Respiratory Medicine, Erasmus MC, Rotterdam, The Netherlands. [25]Centre for Heart Lung Innovation, University of British Columbia, St. Paul's Hospital, Vancouver, BC, Canada. [26]Division of Respiratory Medicine, Department of Medicine, University of British Columbia, Vancouver, BC, Canada. [27]Lothian Birth Cohorts, Department of Psychology, University of Edinburgh, Edinburgh, UK. [28]University of Nottingham and NIHR Nottingham Biomedical Research Centre, Nottingham, UK. [29]Leicester NIHR Biomedical Research Centre, Leicester, UK. [30]Joint senior authors.

Corresponding author: James W. Dodd (james.dodd@bristol.ac.uk)

Check for updates

Shareable abstract (@ERSpublications)
This is the first GWAS to report genome-wide significant SNPs for PRISm, four of which are novel for lung function. Genetic factors associated with PRISm are strongly correlated with risk of both other lung diseases and extrapulmonary comorbidity. https://bit.ly/3Qo0jUn

Cite this article as: Higbee DH, Lirio A, Hamilton F, *et al*. Genome-wide association study of preserved ratio impaired spirometry (PRISm). *Eur Respir J* 2024; 63: 2300337 [DOI: 10.1183/13993003.00337-2023].

This extracted version can be shared freely online.

The content of this work is not subject to copyright. Design and branding are copyright ©ERS 2024.

Abstract

Background Preserved ratio impaired spirometry (PRISm) is defined as a forced expiratory volume in 1 s (FEV_1) <80% predicted and FEV_1/forced vital capacity ⩾0.70. PRISm is associated with respiratory symptoms and comorbidities. Our objective was to discover novel genetic signals for PRISm and see if they provide insight into the pathogenesis of PRISm and associated comorbidities.

This version is distributed under the terms of the Creative Commons Attribution Licence 4.0.

Received: 2 March 2023
Accepted: 29 Oct 2023

Methods We undertook a genome-wide association study (GWAS) of PRISm in UK Biobank participants (Stage 1), and selected single nucleotide polymorphisms (SNPs) reaching genome-wide significance for replication in 13 cohorts (Stage 2). A combined meta-analysis of Stage 1 and Stage 2 was done to determine top SNPs. We used cross-trait linkage disequilibrium score regression to estimate genome-wide genetic correlation between PRISm and pulmonary and extrapulmonary traits. Phenome-wide association studies of top SNPs were performed.

Results 22 signals reached significance in the joint meta-analysis, including four signals novel for lung function. A strong genome-wide genetic correlation (r_g) between PRISm and spirometric COPD (r_g=0.62, p<0.001) was observed, and genetic correlation with type 2 diabetes (r_g=0.12, p=0.007). Phenome-wide association studies showed that 18 of 22 signals were associated with diabetic traits and seven with blood pressure traits.

Conclusion This is the first GWAS to successfully identify SNPs associated with PRISm. Four of the signals, rs7652391 (nearest gene *MECOM*), rs9431040 (*HLX*), rs62018863 (*TMEM114*) and rs185937162 (*HLA-B*), have not been described in association with lung function before, demonstrating the utility of using different lung function phenotypes in GWAS. Genetic factors associated with PRISm are strongly correlated with risk of both other lung diseases and extrapulmonary comorbidity.
